# Types of Nasal Delivery Drugs and Medications in Iranian Traditional Medicine to Treatment of Headache

**DOI:** 10.5812/ircmj.15935

**Published:** 2014-06-05

**Authors:** Zahra Ghorbanifar, Hosein Delavar Kasmaei, Bagher Minaei, Hossein Rezaeizadeh, Farid Zayeri

**Affiliations:** 1 Department of Traditional Medicine, Shahid Beheshti University of Medical Sciences, Tehran, IR Iran; 2 Department of Neurology, Shohada-e-Tajrish Hospital, Shahid Beheshti University of Medical Sciences, Tehran, IR Iran; 3 Department of Traditional Medicine, Tehran University of Medical Sciences, Tehran, IR Iran; 4 Department of Biostatistics, Shahid Beheshti University of Medical Sciences, Tehran, IR Iran

**Keywords:** Headache, Herbal Medicine, Nose, Analgesics

## Abstract

**Context:**

Headache is a common symptom throughout the world. The main purpose of patient-centered approaches is the utilization of useful and simple treatment. Nowadays, there is a rising propensity toward herbal remedies. Nasal route is one of the ancient and topical prescriptions used in headache. In Iranian traditional medicine, physicians such as Avicenna were prescribing herbal drugs through the nose to treat a variety of central nervous system diseases like headache.

In this review paper, authors have attempted to introduce different types of nasal administrations which were used in Iranian traditional medicine for the treatment of headaches.

**Evidence Acquisition:**

Initially, we studied two different types of Canon and separated all herbs used in the treatment of headache. Next, all plants were classified according to the method of prescription. Then, we pick out all the plants which were nasally utilized in the treatment of headache and divided them based on the method of administration. In order to find scientific names of herbs, we used two different botany references. Moreover, we conducted various researches in scientific databases with the aim of finding results concerning the analgesic and antinociceptive effects of herbs. Throughout the research, key terms were “analgesic” and “antinociceptive “with the scientific names of all herbs separately. The databases searched included PubMed, Scopus, Cochrane library and SID.

**Results:**

35 plants were prescribed for the treatment of headaches, which were all nasally used. These plants took either the form of powder, liquid or gas (steam). They were divided in to six categories according to the method of prescription. The Percentage of usage for each method was as follows: 62% Saoot (nasal drop), 25% Shamoom (smell), 17% Inkabab (vapor), 11% Nafookh (snuff), 11% Nashooq (inhaling) and 2% Bokhoor (smoke).

**Conclusions:**

Medications that are used via nasal delivery have greater effect than oral medications. Iranian physicians were fully aware of systemic effects of topical medications, including prescription drugs through the nose. The study of ancient medical texts helps us in identification of herbal medicine and the investigation of new way for the preparation of drugs.

## 1. Context

Headache is an extremely common symptom throughout the world. According to the studies of scientists incidence, prevalence as well as the individual and social costs of headache are significantly high all over the world (1) especially in low and middle-income countries (2). While the prevalence of headache is more than 47% (3) utilization of proper and continuous management in the treatment of headache will reduce the personal and social expenditures of headache disorders (4). Because of the side effects resulting from the oral drugs, physicians tend to use other delivery mechanisms such as nasal delivery administration (5). Nowadays, the main purpose of patient-centered approaches is the identification and development of the most useful and simple treatment that can be acceptable for patients. At the moment, more than 20% of Americans are using herbal therapy (6). According to the decisions of the World Health Organization (WHO), developing countries were encouraged to utilize traditional medicine in situations that modern medicine approach does not have any offer (7). Thus, currently, the world witnesses a rising propensity towards herbal remedies. Statistics shows that over 80% of people in developing countries apply herbal remedies for curative needs (8). Recently, the employment of complementary medicine in patients with headache is increasing. Investigations in Germany and Austria confirmed this claim because 87% of patients with headache in these two countries use complementary medicine (9).

Studies indicate, that nasal inhalation of herbs is effective to treat different types of headaches (10). Based on such studies and according to the strategy of WHO, utilization of ancient but helpful and effective remedies can be advantageous for patients in all countries (11). Historically, medicine is transmitted from past generations to the present ones but the role of earliest civilizations such as Egyptians, Greek, Persian, Indian, and Chinese in forming the nucleus of medicine is very important (12). Investigation about medieval herbal medicine with the purpose of recognizing the most efficient and secure drugs is growing (13). Herbal therapy is not only a main part of complementary and alternative medicine but also it is actually a traditional preventive and curative method (14, 15). History of the herbs used in the treatment of diseases including headache in Iran, goes back to sixth century BC (16). Medieval Iranian practitioners not only knew the medical traditions of ancient Greece, Egypt, India, China and theories of Hippocrates and Galen but they also added to this knowledge, their own detailed experiments and many new scientific theories. The dosage of drugs and the way of administration were very important for Iranian physicians (17), and prescription drugs through the nose is one of the ancient methods of administration (18).

Ebn-e-Sina (980-1037), who is known as Avicenna in the West, was a Persian prominent philosopher and physician. The most notable of his writings is Canon of Medicine (19). This book has five volumes. These volumes are consisting of general principles of medicine, Single medicines, diseases of individual organs and general diseases and pharmaceutical medicine (20). Accordingly, the authors of this paper have attempted to introduce different types of nasal administrations which were used in Iranian traditional medicine for the treatment of headaches.

## 2. Evidence Acquisition

The first step was investigation of two different type of Canon: the translated version of Canon in English (21) and the edited version of Canon in Arabic (22). Throughout our research, we separated all herbs used for the treatment of headache from chapter "Single Medicines” in the first and second volume of Canon and we studied headache section to find all plants that he had prescribed as a treatment for all types of headaches. Therefore, this paper does not concern itself with the animal or mineral sources discussed by Avicenna.

All plants were classified according to the method of prescription. After wards, we pick out all the plants used nasally for the treatment of headache and divided them into categories based on the method of administration. It also became clear that each plant is effective in what kind of headache. In order to facilitate further examination and analysis we organized the results of research on to a Table 1. However, all methods of nasal treatment are explained in the paper. We used botanical textbooks for family and scientific names of the medicinal plants (23, 24). In order to find results concerning the analgesic and antinociceptive effects of separated plants in this paper, we searched the scientific databases. The databases consulted PubMed, Scopus, Cochrane library, SID up to October 11, 2013. The key terms of search were “analgesic” and “antinociceptive” with all scientific names of plants separately. All human and animal studies that included the evidences of analgesic and antinociceptive effects of herbs written in this article were selected for review. Only publications without available full text and letters to the editor were excluded from the review. Unpublished data was also excluded from the study. Duplication was avoided by excluding reviews of multiple copies of the same article in several databases.

## 3. Results

In the single medicines chapter of Canon, Avicenna has introduced about 800 drugs. They include plants, animal products, and minerals but most of them are plants (25). He has described the function of each of them based on several principles:

- the effects of the drug in different organs of the body (positive or negative).
- The Effects of the drug on specific disease in any organ.
- The aim of application (prevention, treatment, nourishing).
- The method of usage (oral or topical).

As was mentioned, these drugs are administered systemically or topically. Topical treatment of headache has a great variety of methods. Prescription of drugs through the nose is one of topical remedies. 35 plants are typically prescribed for nasal usage in order to treat headaches. Moreover, about 80% of topical drugs applied in headache through the nose are herbal.

Plant parts used in the treatment of disease consist of leaves, roots, seeds, flowers, fruits and gums (21). In the treatment of headaches, herbs are used in three forms; powder, liquid (watery or oily solution) and gas or steam. These forms are divided into six categories according to the method of prescription in the treatment of headaches. These six methods consist of: Saoot (nasal drop): Oily or watery drug dropped in the nose. This class of drugs is primarily used orally. In case, the patient is unable to take it orally, the drug is diluted and dropped inside the nose. Nafookh (snuff): Powder is inbreathed in nose through pipe or directly. Shamoom (smell): A medicine, which is smelled. Nashooq (inhaling): It is a watery drug that is only sucked in the nose. Bokhoor (smoke): It is a medicine which is used through its smoke. Inkabab (vapor): Some drugs are boiled and their vapors are inhaled to nose (26).

Some plants are prescribed just through one method. But a numbers of other plants are applied via several methods. Accordingly, percentage of utilization from variety of methods is as follows: 62% Saoot, 25% Shamoom, 17% Inkabab, 11% Nafookh, 11% Nashooq and 2% Bokhoor. The other point is that herbal drugs are used alone or in combination with other materials like milk, vinegar or some oils. For example, oil of Ecballium elaterium L (Ghesa-al-hemar) must be used with milk. Myristica fragrans Houtt (Basbaase) is mixed with Viola odorata oil after that it can be used in the form of saoot. Additionally, in order to use Cinnamomum camphora (Kaafoor) in the form of shamoom, it must be mixed with lettuce oil. The purpose of these procedures is the reduction of drugs side effects and the decrease of the time it takes for the drug, to reach the brain (22).

**Table 1.** Types of Herbs and How to Use Them Via the Nose to Treatment of Headache^a^

Plant Family	Scientific Name	Persian Name	Type of Administration	Plant Parts Used	Type of Headache	Activity
***Violaceae***	*Viola odorata L.*	Banafsaj	Saoot	Oil	Warm, cold and Helmet headache	A (27)
			Shamoom	Flower	Headache caused by stench	
			Inkebab	Flower	Headache caused by stench	
			Nashooq	Flower	Congestive headache	
***Rosaceae***	*R .damascena Mill.*	Vardahmar	Saoot	Oil	Warm headache, Headache caused by dense gases	A (28)
			Shamoom Inkebab Nashooq	Oil-Water	Headache caused by stench	
***Ranunculaceae***	*Nigella sativa*	Shooneez	Saoot	Seed	Cold headache	A, AN (29)
			Nafookh	Seed	Chronic headache	
***Lamiaceae***	*Zataria multiflora Boiss.*	Satar	Saoot	Leaves	Cold headache	AN (30)
***Brassicaceae***	*Brassica nigra L.*	Khardal	Saoot	Seed	Cold headache	A (31)
***Styracaceae***	*Styrax officinalis L.*	Meiey	Saoot	Gum	Cold headache	-
***Cucurbitaceae***	*Citrullus colocynthis (L.) Schrad*	Hanzal	Saoot	Oil	Cold headache	A (32)
***Cucurbitaceae***	*Ecballium elaterium L.*	Ghesa-al-hemar	Saoot	Oil	Cold, migraine, Chronic and helmet headache	
***Fabaceae***	*Alhagi maurorum Medik*	Haaj	Saoot	Leaves	Cold headache	A (33)
***Rutaceae***	*Ruta graveolens*	Saafsia	Saoot	Gum	Cold headache	
			Nafookh	Gum	Chronic headache	
***Liliaceae***	*Lilium speciosum Thunb*	Soosan	Saoot	Root	Cold headache	-
***Euphorbiaceae***	*Euphorbia resinifera A. erger*	Farbiyoon	Saoot	Gum	Cold headache	-
***Amaranthaceae***	*Beta vulgaris L.*	Salgh	Saoot	Extract of root	Cold headache	AN (34)
***Berberidaceae***	*Berberis aristata*	Hozazhendi	Saoot	Extract	Cold headache	-
***Anacardiaceae***	*Pistacia vera L.*	Fostogh	Saoot	Oil	Unilateral headache	A (35)
***Asteraceae***	*Matricaria chamomilla L.*	Baaboonaj	Saoot	Oil	Cold headache	A (36)
***Apiaceae***	*Peucedanumgraveolens L.*	Shebet	Saoot	Oil	Headache caused by dense gases	-
***Lythraceae***	*Lawsonia inermis L.*	Hanaa	Saoot	Oil	Cold headache	-
***Rosaceae***	*Amigdalus communis L.*	Loz	Saoot	Oil	Dry headache-Throbbing headache	-
***Primulaceae***	*Cyclamen europaeum L.*	Bokhurmaryam	Nafookh	-	Cold and Chronic headache	-
***Anacardiaceae***	*Rhus coriaria L.*	Somaagh	Nafookh	Seed	Cold headache	-
***Lamiaceae***	*Origanum majorana L.*	Marzanjush	Shamoom	Leave	Headache caused by dense gases	AN (37)
***Nymphaeaceae***	*Nymphaea alba L.*	Niloofar	Shamoom	Flower	Headache caused by stench	-
***Lauraceae***	*Cinnamomum camphora L.*	Kaafoor	Shamoom	-	Headache from warm smells-helmetheadache	A (38)
			Inkebab	-	Headache caused by stench	
***Santalaceae***	*Santalum album L.*	Sandal	Shamoom	Wood	Headache caused by warm smells	-
***Iridaceae***	*Crocus sativus L.*	Zafaraan	Shamoom	Flower	Headache caused by cold smells	AN (39)
***Asterceae***	*Lactuca sativa L.*	khas	Inkebab	Oil	Headache caused by stench	A (40)
***Salicaceae***	*Salix caprea L.*	Khelaaf	Inkebab Nashooq	Flower and juice	Headache caused by stench	-
***Cucurbitaceae***	*Cucurbita pepo L.*	Ghar	Inkebab Nashooq	Seed, oil	Headache caused by stench	-
***Myristicaceae***	*Myristica fragrans Houtt*	Basbaase	Saoot	Peel	Migraine, Headache caused by dense gases	A (41)
***Taxaceae***	*Taxus baccata L.*	Zarnab	Saoot	-	Cold headache	AN (42)
***Apiaceae***	*Pimpinella anisum L.*	Anisoon	Bokhoor	Seed	General	A (43)
***Lamiaceae***	*Teucrium montanum L.*	Marmaahooz	Shamoom	-	Cold headache	-
***Oleaceae***	*Jasminum officinale L.*	Yaasamin	Shamoom	Flower	Phlegmatic headache	AN (42)
***Spanidaceae***	*Sapindus trifolius L.*	Rateh	Saoot	Fruit	General, unilateral headache	AN (44)

^a^ Abbreviations: A, analgesic; AN, antinociceptive.

## 4. Conclusions

Utilization of systemic medications for the treatment of headache has some problems. Blood-brain barrier is an obstacle for treatment of central nervous system diseases. Endothelial junctions of blood-brain barrier are too close. These junctions are about 100 times closer than other capillary endothelium (45). Moreover gastrointestinal symptoms such as nausea and vomiting with headache are significant obstacles to the use of oral medications. Recent clinical studies indicate that the medications which are used via nasal delivery, powder or spray form, have greater effect than oral medications (46). Thus prescription of drugs through the nose is one of the ways to dominate these barriers.

Nose is an alternative route for drug delivery because endothelial membrane of nasal mucosa is spongy and its blood flow is high. Moreover, the surface area of two nasal cavities is about 150 cm^2^. In central nervous system diseases such as pain, fast and proprietary drug delivery is required and necessary. In such situations, nasal delivery is an appropriate method (47). This method has many advantages, for example: absorption of drugs is rapid and beginning of the action is fast. Not only is there, no hepatic metabolism and gastrointestinal degradation of drugs, but also there is high bioavailability of drugs. Compared with intravenous method during prolonged therapy, it is an alternative treatment (48). Currently, some headache medications are administered via nasal spray or in the powder form. Utilization of nasal spray after 15 minutes relieves headache. Among headache medicines, Triptans and NSAIDs have nasal form (49). As a result, Iranian traditional medicine maybe a good source for research on medicinal plants. Iranian physicians such as Avicenna were fully aware of systemic effects of topical medications, including prescription drugs through the nose. They used this method to treat a variety of central nervous system diseases like headache. Based on clinical and pharmacological studies about mentioned plants in this paper, half of them have analgesic or antinociceptive effect. Hence, utilization of herbs whose their safety has been confirmed by the clinical studies can be effective and efficient in the treatment of headache.

It seems that research on traditional medicine about other plants as well as other effective therapies in headache help us to recognize simple remedies and may lead to better treatment of patients. Medications that are used via nasal delivery have greater effect than oral medications. Iranian physicians were fully aware of systemic effects of topical medications, including prescription drugs through the nose. The study of ancient medical texts helps us in identification of herbal medicine and the investigation of new way for the preparation of drugs.
